# Supplementary figures and images for: Mistargeted retinal axons induce a synaptically independent subcircuit in the visual thalamus of albino mice
Source: bioRxiv. 2024 Dec 3:2024.07.15.603571. Originally published 2024 Jul 17. Preprint. [Version 2] doi: 10.1101/2024.07.15.603571 (PMC11275878; doi:10.1101/2024.07.15.603571)

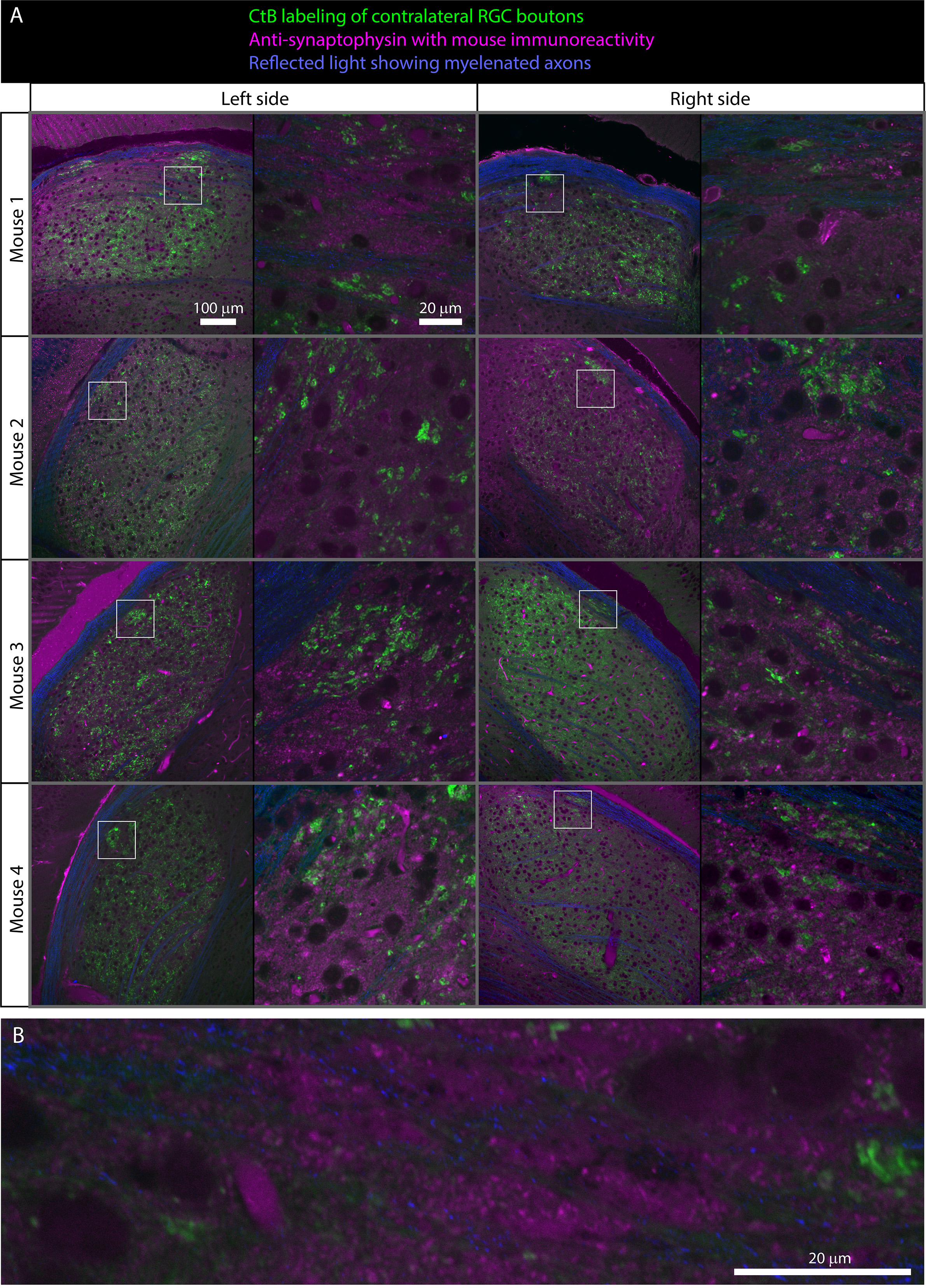

Supplement: Supplement 1 — Supplemental Figure 1. Synaptophysin labeling of dLGN synapses shows synaptic neuropil is present in the RGC bouton exclusion zone. A) Each row show the left and right dLGN is shown for each mouse. Contralateral projecting RGC terminals labeled with CtB (488 or 555) are shown in green. Mouse anti-synaptophysin immunoreactivity is shown in magenta. Anti-mouse secondary antibody labels blood vessels as well as anti-synaptophysin primary antibody. Reflected light highlighting myelinated axons is shown in blue. A) For each dLGN, as single plane of a confocal scan of the full dLGN (left) and exclusion zone (right) is shown. The white box indicates the position of the high-resolution image relative to the full dLGN. Bright punctate immune artifacts are visible in some panels. B) Closer look at exclusion zone of left dLGN from mouse 1 shown in panel A. The immunolabeling showing synaptic neuropil in the exclusion zone is consistent with our examination of the EM volume. [file media-1.jpg]

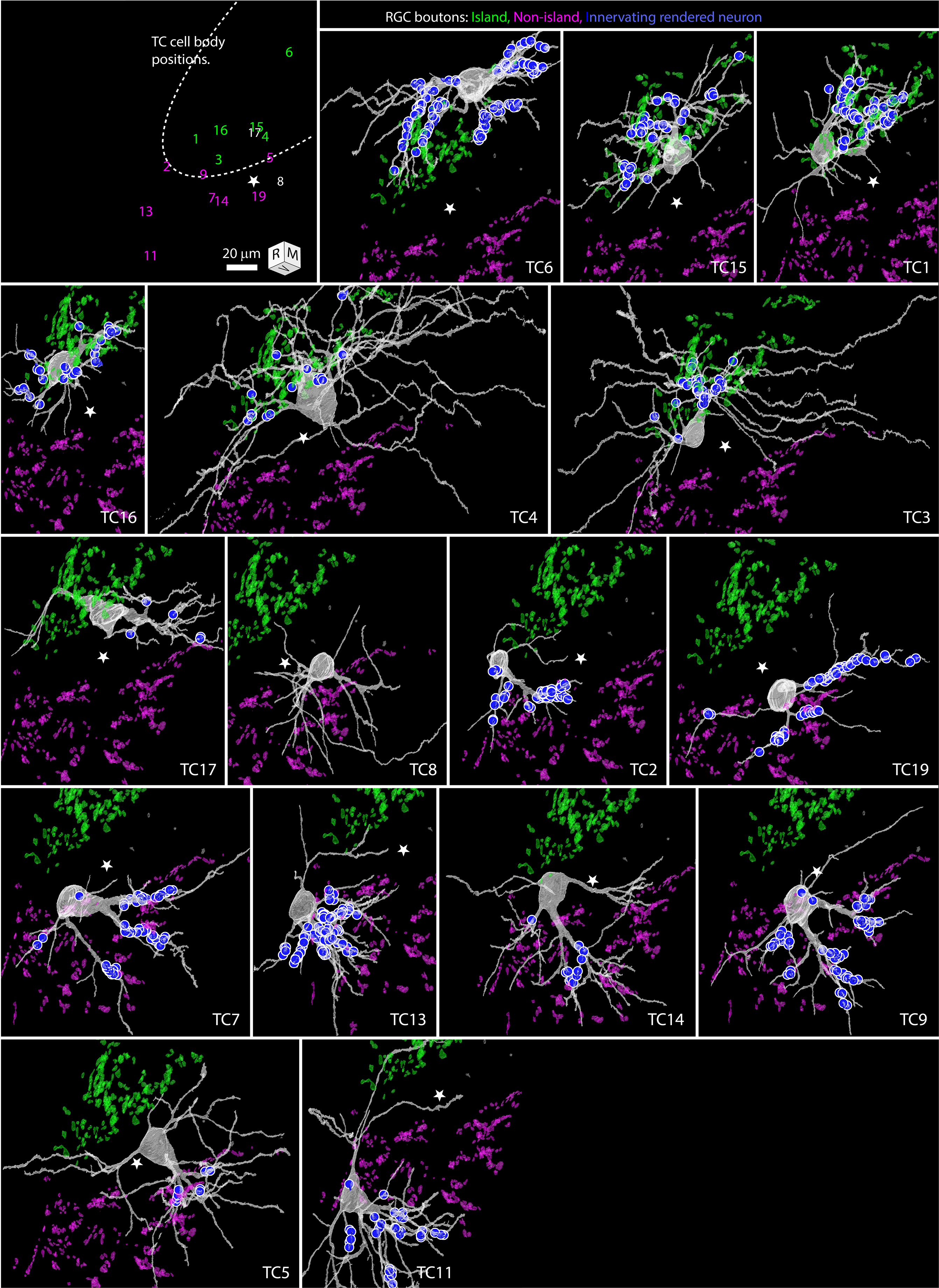

Supplement: Supplement 2 — Supplementary Figure 2. TCs surrounding the exclusion zone are captured by either the island or non-island RGC boutons. The TC dendritic arbors reflect this capture. Panels show renderings of partially reconstructed TCs. The top left key shows the position of the nuclei of the reconstructed TCs relative to the boundary of the RGC island (dotted white line). The color of the cell IDs indicates whether they received RGC input from the island or non-island RGC boutons. The star indicates a common reference position for all panels. RGC inputs innervating the TC highlighted in each panel are shown as blue circles. The TC connectivity and morphologies are consistent with Hebbian rules shaping retinogeniculate circuit structure. [file media-2.tif]

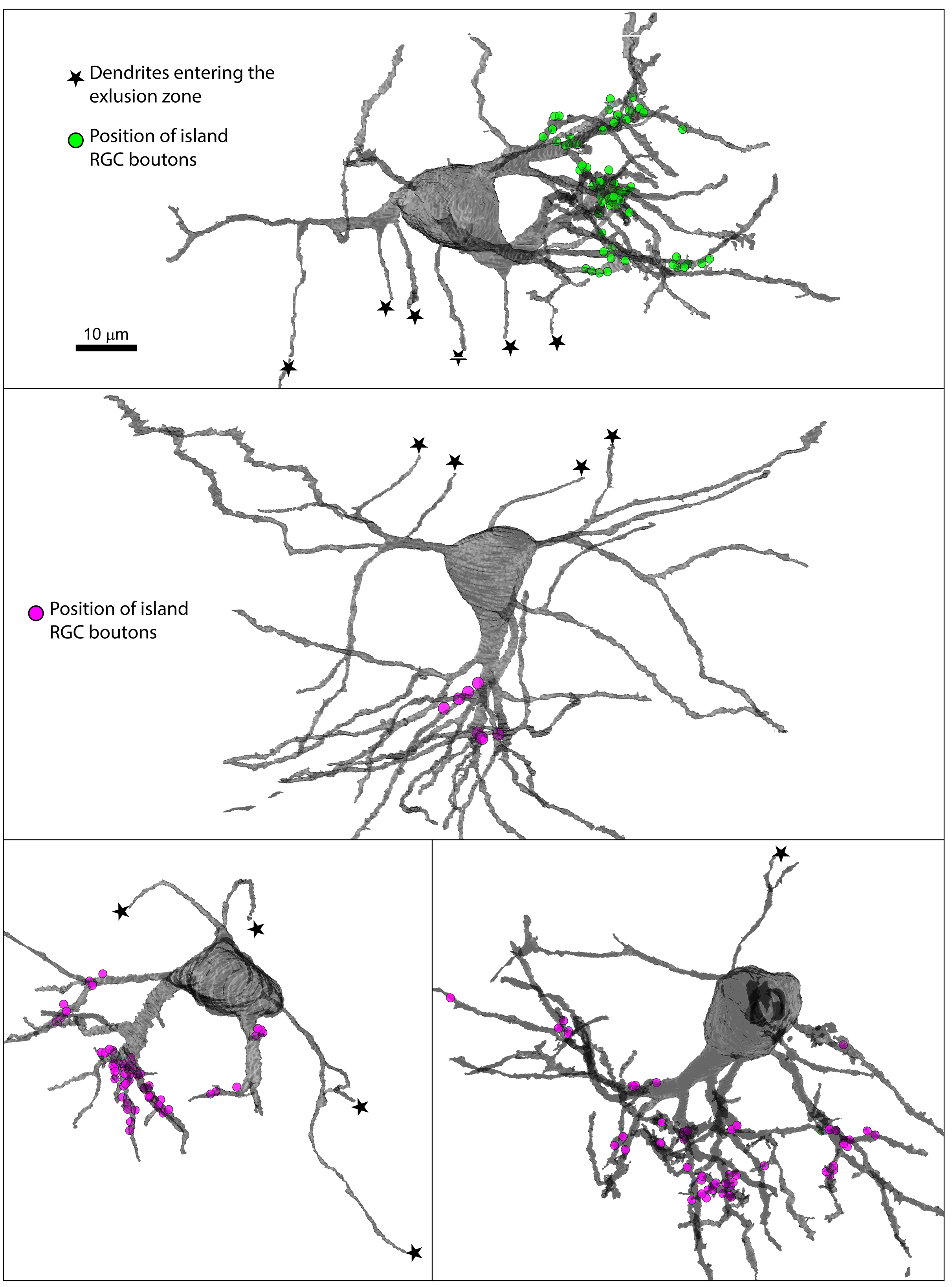

Supplement: Supplement 3 — Supplementary Figure 3. Proximal dendrites of TCs are usually thick and innervated by RGC boutons. Proximal dendrites of TCs that extend into the exclusion zone are noticeably thinner, similar to distal dendrites. Panels show four example TCs where dendrites were observed extending into the exclusion zone. Neurites in the exclusion zone are indicated with stars. The location of RGC boutons innervating the TC are indicated by circles (green = island, magenta = non-island). [file media-3.tif]
