# Supplementary material for: Mistargeted retinal axons form synaptically segregated subcircuits in the visual thalamus of albino mice
Source: bioRxiv. 2024 Jul 17:2024.07.15.603571. Preprint. [Version 1] doi: 10.1101/2024.07.15.603571 (PMC11275878; doi:10.1101/2024.07.15.603571)
Supplement: Supplementary file 1 [file NIHPP2024.07.15.603571V1-supplement-1.pdf]

bioRxiv preprint doi: https://doi.org/10.1101/2024.07.15.603571; this version posted July 17, 2024. The copyright holder for this preprint (which was not certified by peer review) is the author/funder, who has granted bioRxiv a license to display the preprint in perpetuity. It is made available under aCC-BY 4.0 International license.

SUPPLEMENTAL

bioRxiv preprint doi: https://doi.org/10.1101/2024.07.15.603571; this version posted July 17, 2024. The copyright holder for this preprint (which was not certified by peer review) is the author/funder, who has granted bioRxiv a license to display the preprint in perpetuity. It is made available under aCC-BY 4.0 International license.

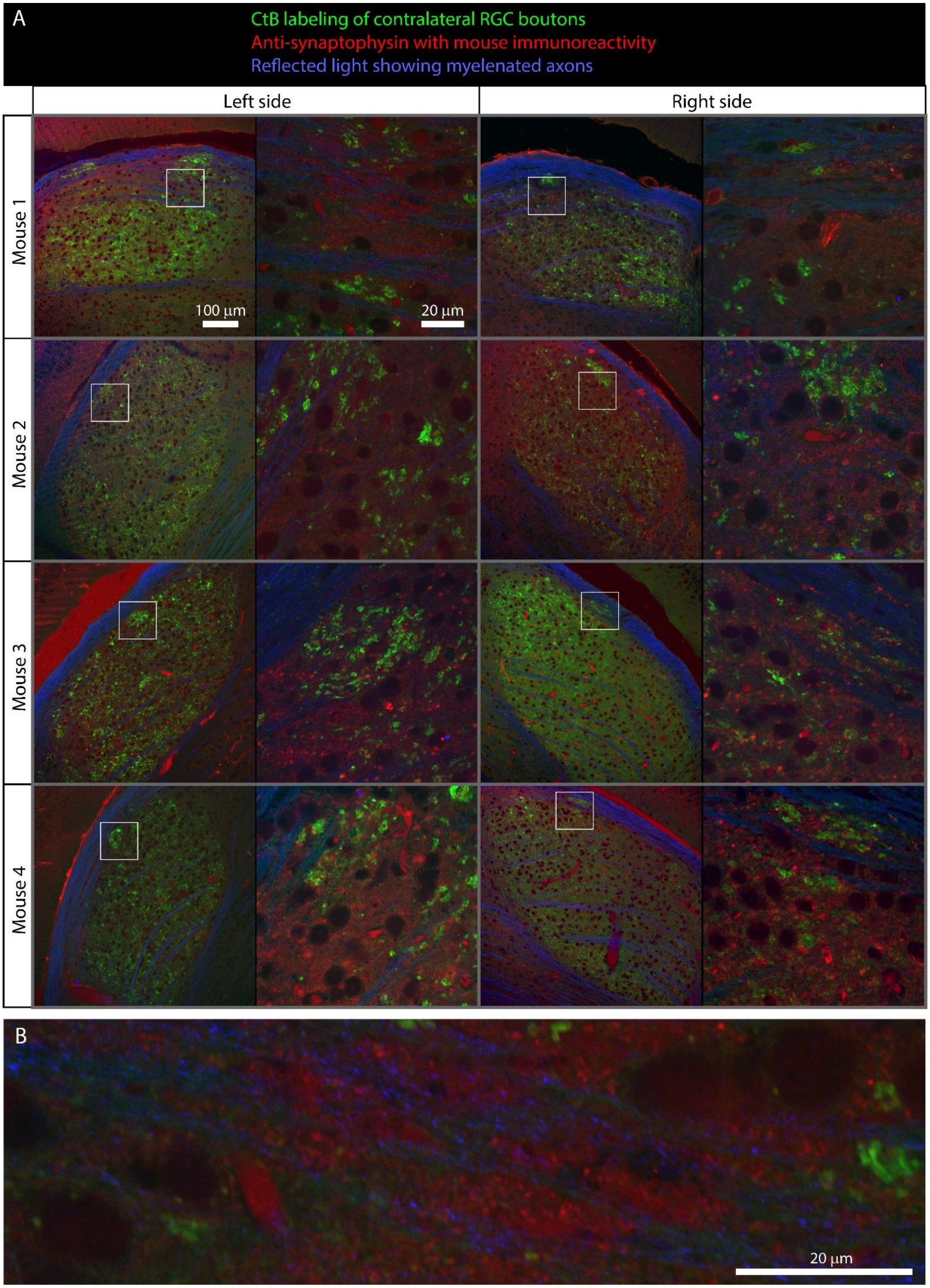

bioRxiv preprint doi: https://doi.org/10.1101/2024.07.15.603571; this version posted July 17, 2024. The copyright holder for this preprint (which was not certified by peer review) is the author/funder, who has granted bioRxiv a license to display the preprint in perpetuity. It is made available under aCC-BY 4.0 International license.

Supplemental Figure 1. Examination of the exclusion zone of four albino mice. Left and right dLGN is shown for each mouse. Contralateral projecting RGC terminals labeled with CtB (488 or 555) are shown in green. Mouse anti-synaptophysin immunoreactivity is shown in red. Anti-mouse secondary antibody labels blood vessels as well as anti-synaptophysin primary antibody. Reflected light highlighting myelinated axons is shown in blue. A) For each dLGN, as single plane of a confocal scan of the full dLGN (left) and exclusion zone (right) is shown. The white box indicates the position of the high-resolution image relative to the full dLGN. Bright punctate immune artifacts are visible in some panels. B) Closer look at exclusion zone of left dLGN from mouse 1 shown in panel A.

bioRxiv preprint doi: https://doi.org/10.1101/2024.07.15.603571; this version posted July 17, 2024. The copyright holder for this preprint (which was not certified by peer review) is the author/funder, who has granted bioRxiv a license to display the preprint in perpetuity. It is made available under aCC-BY 4.0 International license.

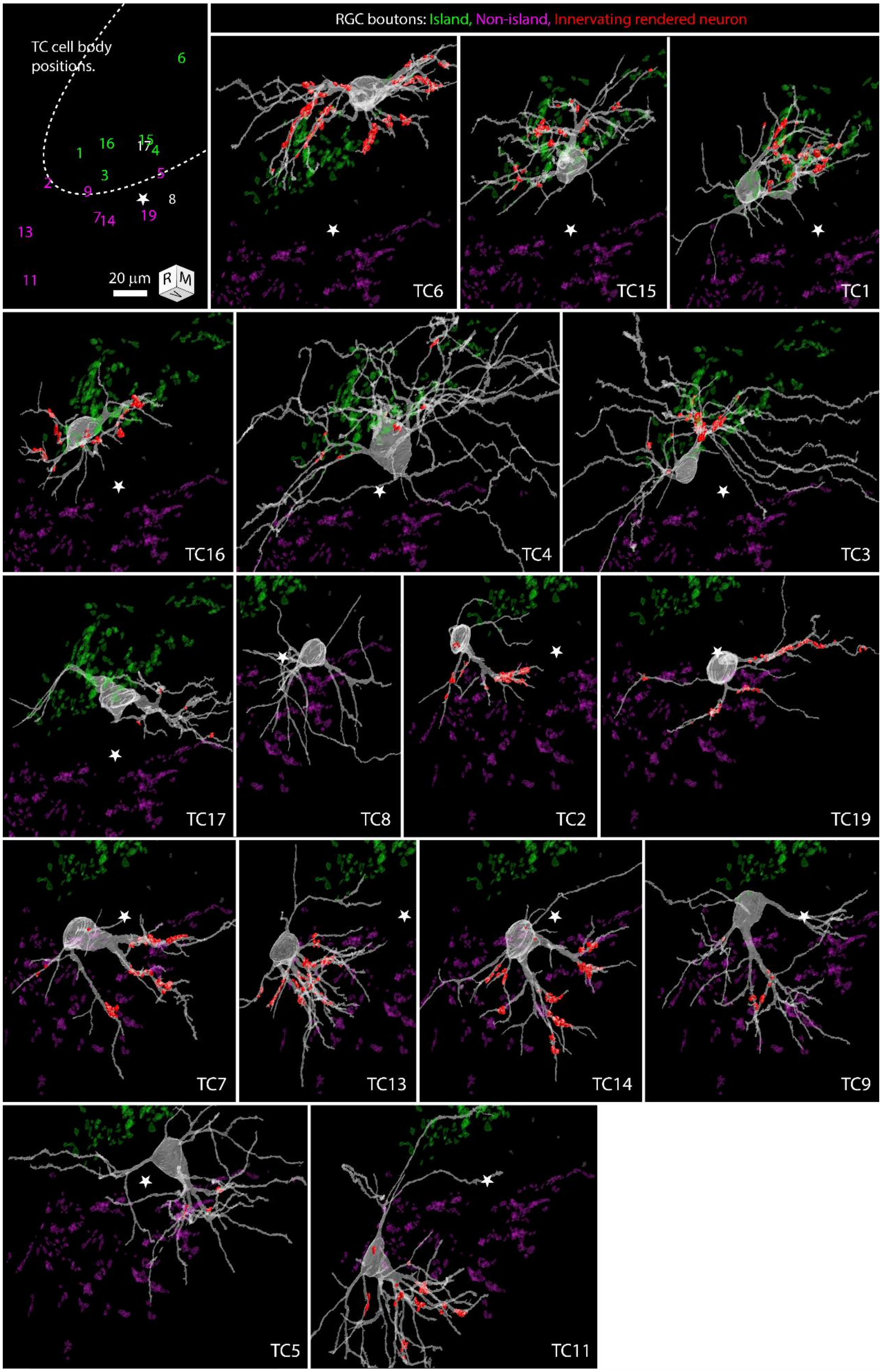

bioRxiv preprint doi: https://doi.org/10.1101/2024.07.15.603571; this version posted July 17, 2024. The copyright holder for this preprint (which was not certified by peer review) is the author/funder, who has granted bioRxiv a license to display the preprint in perpetuity. It is made available under aCC-BY 4.0 International license.

Supplementary Figure 2. Renderings of partially reconstructed TCs. The top left key shows the position of the nuclei of the reconstructed TCs relative to the boundary of the RGC island (dotted white line). The color of the cell IDs indicates whether they received RGC input from the island or non-island RGC boutons. The star indicates a common reference position for all panels. RGC inputs innervating the TC highlighted in each panel are shown in red.

bioRxiv preprint doi: https://doi.org/10.1101/2024.07.15.603571; this version posted July 17, 2024. The copyright holder for this preprint (which was not certified by peer review) is the author/funder, who has granted bioRxiv a license to display the preprint in perpetuity. It is made available under aCC-BY 4.0 International license.

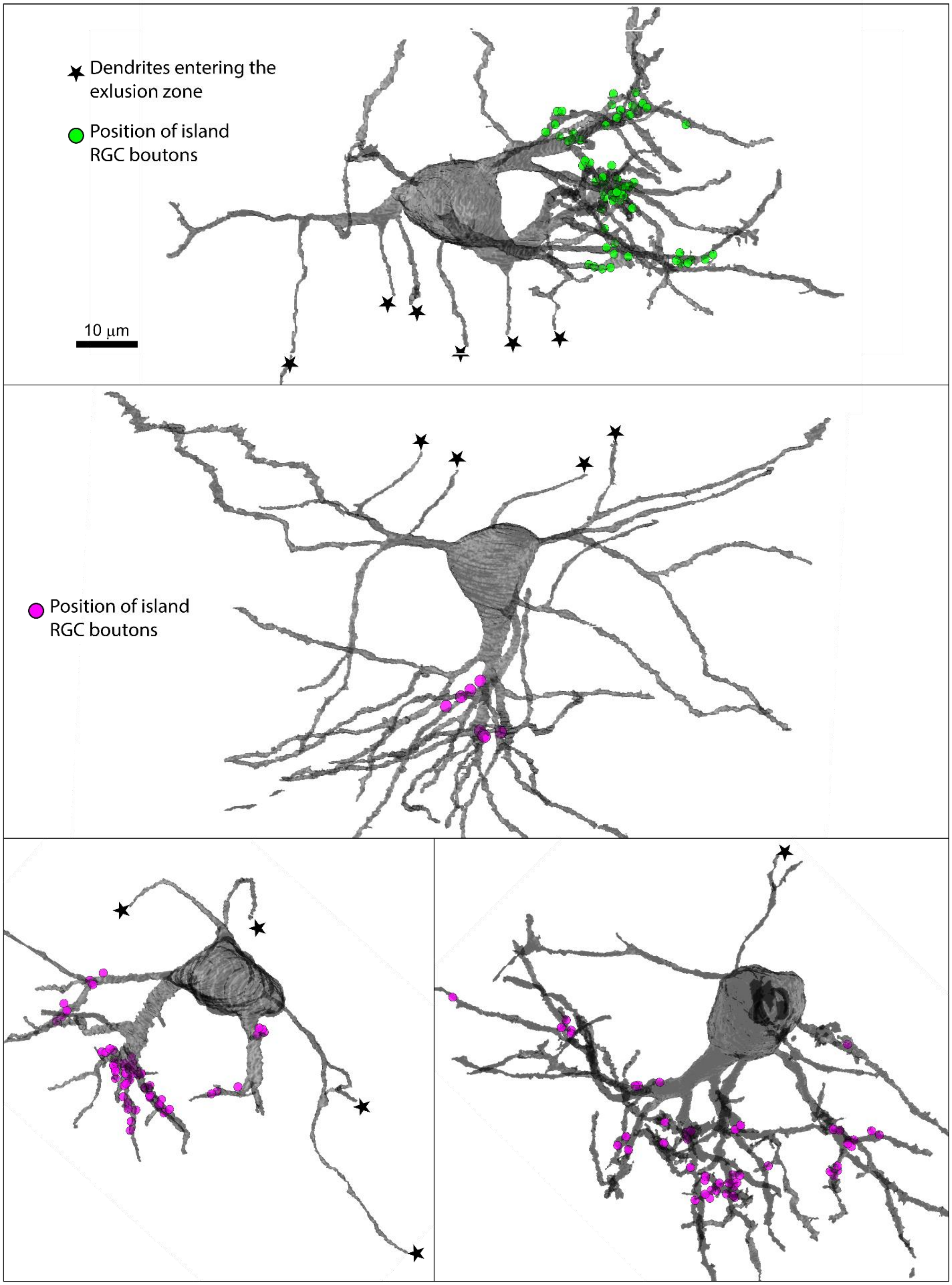

bioRxiv preprint doi: https://doi.org/10.1101/2024.07.15.603571; this version posted July 17, 2024. The copyright holder for this preprint (which was not certified by peer review) is the author/funder, who has granted bioRxiv a license to display the preprint in perpetuity. It is made available under aCC-BY 4.0 International license.

Supplementary Figure 3. Proximal dendritic arbors of four example TCs where thin dendrites extending into the exclusion zone were observed. Neurites in the exclusion zone are indicated with stars. The location of RGC boutons innervating the TC are indicated by circles (green = island, magenta = non-island).
